# 4.L. Round table: Mobilising coping and support strategies: learning from healthcare workers’ experiences of COVID-19

**DOI:** 10.1093/eurpub/ckac129.251

**Published:** 2022-10-25

**Authors:** Organised by: Robert Koch-Institute, EUPHA-HWR, Chair persons: Heide Weishaar (Germany), Ellen Kuhlmann (EUPHA-HWR)

## Abstract

**Background:**

During the COVID-19 pandemic, health care workers (HCWs) have experienced considerable stress and shown great resilience. Multiple organisations advocate for better support for the health workforce, both during pandemics and in routine health care. Effective interventions aimed at supporting HCWs should be based on evidence stemming from HCWs’ coping strategies and support needs. This workshop highlights coping mechanisms and support strategies for HCWs during public health emergencies. It is chaired by the Robert Koch Institute (RKI), Germany's national public health institute, who played a major role in the pandemic response over the last two years, and the EUPHA Health Workforce Research Section. While the workshop's primary geographical focus is Germany, the roundtable participants will explore, from an international perspective, what lessons can be learned from HCWs’ experiences during the COVID-19 pandemic and discuss the relevance to researchers, health professionals, health managers, politicians, and other decision makers.

**Objectives:**

The aims are to: (i) introduce an analytical framework for coping mechanisms and support strategies for HCWs during public health emergencies, (ii) present reported coping mechanisms among, and support strategies provided to, HCWs during the COVID-19 pandemic, and (iii) discuss the coping mechanisms and support strategies that have been reported to be most effective in improving the situation of HCWs in the future.

The panel workshop starts with two 10-minute presentations followed by a moderated panel and subsequent discussion with the audience. The presentations introduce the workshop topic using a scoping literature review conducted at RKI on HCWs’ experiences during the COVID-19 pandemic, and a summary of key findings of a mixed-method study on coping strategies and support needs of HCWs during COVID-19 in Germany, highlighting support needed and received and crucial sources of support. The panel highlights the most effective reported coping mechanism, addresses gaps in support strategies, and collects suggestions for how to make use of the suggested solutions. These as well as lessons to be learned for policy and practice will be discussed with panel members and attendees, connecting the perspectives of health policymakers, management. and professionals. The aim of the workshop is to contribute to the wellbeing of HCWs, who represent one of the most important pillars of a health system.

**Key messages:**

• Healthcare workers’ coping mechanisms and support strategies during the COVID-19 pandemic are an important source of health workforce resilience.

• Lessons learned from the COVID-19 pandemic on supporting health care workers must be jointly implemented across sectors.

**Speakers/Panellists:**

**Megan Evans**

Robert Koch Institute, Berlin, Germany

**George Valiotis**

European Health Management Association, Brussels, Belgium

**Bernhard Gibis**

Kassenärztliche Bundesvereinigung, Germany

**Véronique S Grazioli**

Center for Primary Care and Public Health, University of Lausanne, Lausanne, Switzerland

**Ber Oomen**

European Specialist Nurses Organisation, Brussels, Belgium

